# Improving pain management for murine orthotopic xenograft models of acute lymphoblastic leukemia

**DOI:** 10.1038/s41684-025-01615-3

**Published:** 2025-10-08

**Authors:** Tim Schreiber, Sandra Lange, Steven R. Talbot, Jakob Brandstetter, Emily Leitner, Christian Junghanss, Brigitte Vollmar, Rupert Palme, Anna Richter, Simone Kumstel

**Affiliations:** 1 Rudolf-Zenker-Institute of Experimental Surgery, University Medical Center Rostock, Rostock, Germany; 2 Department of Medicine, Clinic III – Hematology, Oncology, Palliative Medicine, University Medical Center Rostock, Rostock, Germany; 3 Hannover Medical School, Institute for Laboratory Animal Science, Hannover, Germany; 4 Unit of Experimental Endocrinology, Department of Biological Sciences and Pathobiology, University of Veterinary Medicine, Vienna, Austria

**Keywords:** Cancer models, Animal physiology

## Abstract

Despite ongoing research, realistic in vitro models for acute lymphoblastic leukemia (ALL) that can mimic the complex pathology are still not available, highlighting the need for continuous animal-based investigation. As part of the 3R principles, constant refinement of animal experiments is mandatory. Therefore, reviewing the effectiveness of used analgesics is essential for animal model-specific refinement. Here we evaluate whether metamizole—previously used in our institute—or tramadol is more suitable as on-demand analgesia in mouse models of ALL. The murine orthotopic xenograft models were induced by intravenous injection of either the human precursor ALL cell lines RS4;11 or SEM into immune-deficient male and female NSG mice. Mice were weighed and checked daily for basic behavior and well-being, while detailed welfare parameters, such as burrowing behavior, nesting activity, perianal temperature, liquid intake, fecal corticosterone metabolites, mouse grimace scale and tumor cell proliferation were monitored weekly. Upon leukemic progression, when signs of pain or discomfort were observed, metamizole (3 mg/ml) or tramadol (1 mg/ml) was administered via drinking water for analgesic treatment, and detailed welfare parameters were assessed daily. Following the initiation of treatment, mice receiving either metamizole or tramadol continued to show a decline in body weight, liquid intake and other welfare parameters, suggesting that neither drug was sufficient to fully counteract the effects of late-stage ALL. Combining the data with the relative severity assessment algorithm revealed that metamizole treatment appeared less effective than tramadol in mitigating the detrimental effects of the disease. Therefore, the opioid tramadol should replace metamizole as the analgesic compound of choice for hematological xenograft models to improve animal welfare in future studies.

## Abstract (web summary)

By assessing different clinical, behavioral and welfare parameters, the authors demonstrate that the oral administration of tramadol is more suitable than metamizole as on-demand analgesia in mouse models of acute lymphoblastic leukemia.

## Main

The uncontrollable proliferation of immature lymphocytes and high numbers of these cells in the peripheral blood characterize acute lymphoblastic leukemia (ALL). The survival rate for ALL has increased dramatically over the past four decades, especially in children, whose survival rates now exceed 90% (refs. ^1–3^). Animal models have played a critical role in the development of new therapeutic approaches, such as hematopoietic stem cell transplantation^4^. However, in adults (≥40 years), ALL is still associated with a poor prognosis, with a 5-year survival rate below 25% (refs. ^5,6^). Ongoing research using in vitro methods continuously identifies molecular characteristics and mechanisms; however, translational studies investigating potential therapeutic options are hampered by the inability to cultivate primary patient material in an in vitro setting that can imitate human physiology. Therefore, suitable animal models are still necessary to evaluate new treatment strategies for ALL.

The injection of human leukemic blasts into the tail vein of immune-deficient mice is the commonly used orthotopic approach for a preclinical model of leukemia^7,8^. Continuous refinement of animal models is prescribed by law to reduce pain, distress or lasting harm to the mice^9,10^. Leukemia progression might be associated with bone pain^11–13^ or neuropathic pain^14,15^ that is caused by chemotherapeutic interventions in humans and probably also affects animals. Because pain influences immune response, wound healing and tumor progression, optimal pain management is essential not only for improving animal welfare but also for ensuring the quality of scientific data^16^. The evaluation of an optimal analgesic treatment for this animal model is an important aspect of sustainably improving animal welfare and the quality of the study outcome in future experiments. Set doses of analgesics can be applied by subcutaneous or intraperitoneal injections. However, small mammals such as mice have high metabolic rates, and the analgesic effect lasts no longer than 2–12 h after single-dose administration, depending on the analgesic compound^17^. Further, repeated injections also cause additional distress in mice. The administration of analgesics via drinking water is commonly used to ensure continuous pain relief without unnecessary potentially stressful interventions^18–20^. The orthotopic xenograft model for ALL is well established in our institute. Standard care for this animal model includes continuous provision of wet food, daily monitoring of welfare, ample nesting material to maintain body temperature and on-demand analgesic administration of metamizole in the drinking water.

Metamizole (dipyrone) is regarded as the most potent non-opioid analgesic, with antipyretic and spasmolytic effects^21^. Its mechanism is not entirely understood but relies partially on COX-3 inhibition^21^. Furthermore, metamizole is the third most commonly used drug for postoperative analgesia in mice and rats in Germany for animal research^22^. Metamizole has been shown to be an effective pain medication in animal models of sepsis^23^. However, reduction of body weight observed in some mice after metamizole administration indicates the need to refine pain management in this animal model. Tramadol was chosen as the analgesic of choice for potential refinement because it has demonstrated analgesic effectiveness both after subcutaneous injection in a murine bone cancer model^24^ and after voluntary oral intake via drinking water in animal models for pancreatitis, osteotomy and colitis^18–20^. While a single oral dose or injection of tramadol results in a short plasma half-life of 2–6 h, continuous administration via drinking water allows for sustained high bioavailability^25^.

The present study compared the relative impact of previously used metamizole and tramadol as oral on-demand analgesics in the murine ALL xenograft model. This study assessed sensitive clinical, behavioral and biochemical parameters during leukemia progression to compare directly the analgesic effect of both analgesics. Body weight change, distress score, daily liquid intake, mouse grimace scale (MGS) score^26^ and perianal temperature^27^ were analyzed to assess the clinical state of the mice during leukemia progression and analgesic treatment. Changes in the natural behavioral traits of burrowing^28^ and nesting^29^ were also investigated, and corticosterone metabolites in the feces were quantified for characterization of the hormonal stress response^30^. The most informative parameters (body weight, water intake, distress score, nesting and MGS) were combined into a single score by the relative severity assessment (RELSA) algorithm to directly compare the benefits of these two analgesics in the ALL model^31^. The progression of leukemia was quantified once a week by bioluminescence imaging and measurement of the blast frequency in the blood.

## Results

### Experimental design

**Fig. 1:**
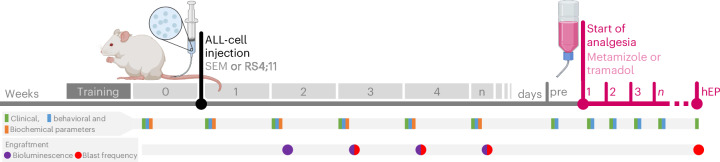
Experimental design of the distinct ALL models with on-demand analgesic treatment. ALL was induced by intravenous injection of either SEM or RS4;11 cells into NSG mice. The assessment of clinical, behavioral and hormonal parameters started before tumor cell injection (week 0) and continued once a week during ALL progression. On-demand analgesic treatment with either metamizole or tramadol began as soon as criteria such as more than 10% body weight loss were met. Afterward, the welfare of the mice was assessed daily by clinical and behavioral parameters until the individual humane endpoint (hEP) of each mouse was reached. The engraftment of leukemic blast was monitored weekly during ALL progression, by bioluminescence imaging and measurement of blast frequency in the blood. Created with BioRender. Kumstel, S. (2025) https://BioRender.com/r3xmnos.

The welfare assessment by clinical, behavioral and biochemical parameters started in the week before leukemic blast injection in healthy NOD.Cg-*Prkdc*^*scid*^ *Il2rg*^*tm1Wjl*^/SzJ mice (NSG) mice (Fig. 1). After injection of either human RS4;11 or SEM cells, the monitoring of the animals’ well-being was performed once a week. Bioluminescence imaging or the analysis of blast frequency in the blood quantified leukemic blast engraftment weekly. At the end of leukemic progression, as soon as criteria such as body weight loss >10%, blast frequency in the blood >20%, squeaking due to pain or abnormal posture were recognized, the mice were randomly assigned into the different analgesic groups. During the continuous administration of analgesics via drinking water, welfare assessment was performed daily until reaching the humane endpoint criteria such as >30% blast frequency in the blood or >20% body weight loss (Fig. 1).

### Analysis of animal’s well-being during leukemia progression

The median survival after injection of the slowly proliferating human RS4;11cells (45 days) was significantly longer than for mice injected with the SEM cells (31 days) (*P* < 0.0001, log-rank (Mantel–Cox) test; Supplementary Fig. 1a). Before injection of the cells and during the progression of leukemia, the welfare of the animals was quantified by sensitive welfare parameters once a week. Impairment of animal welfare was observed, as indicated by a significant reduction in body weight (RS4;11: *P* = 0.0041, repeated measures one-way analysis of variance (ANOVA); SEM: *P* = 0.0054, mixed-effects analysis) during the last week of leukemia progression for both cell lines, compared with healthy mice before tumor cell injection in the first week of the experiment (Supplementary Fig. 1a–c). Furthermore, a significant decrease in liquid intake was seen during the last week of leukemia progression for RS4;11 cells (*P* = 0.0004, Friedman test), and a significant drop in perianal temperature was observed for SEM cell-injected mice (*P* = 0.0108, mixed-effects analysis; Supplementary Fig. 1d–i). A significant increase in the MGS score was noticed during the last 2 weeks of leukemia for both cell lines (RS4;11: *P* < 0.0001, Friedman test; SEM: *P* = 0.0027, mixed-effects analysis; Supplementary Fig. 1j,k).

In addition, a significant reduction of burrowing behavior after 2 h (*P* = 0.0008, Friedman test) and 17 h (*P* = 0.0048, Friedman test) was quantified for the RS4;11 cell-injected mice at the end of leukemic blast engraftment compared with healthy mice during the first week of the experiment (Supplementary Fig. 2a–d). Nesting activity was not significantly changed for both cell lines during leukemia progression, while a nonsignificant increase of fecal corticosterone metabolites (FCMs) was detected (Supplementary Fig. 2e–h).

### Influence of on-demand analgesia on the mouse welfare in the different ALL models

**Fig. 2:**
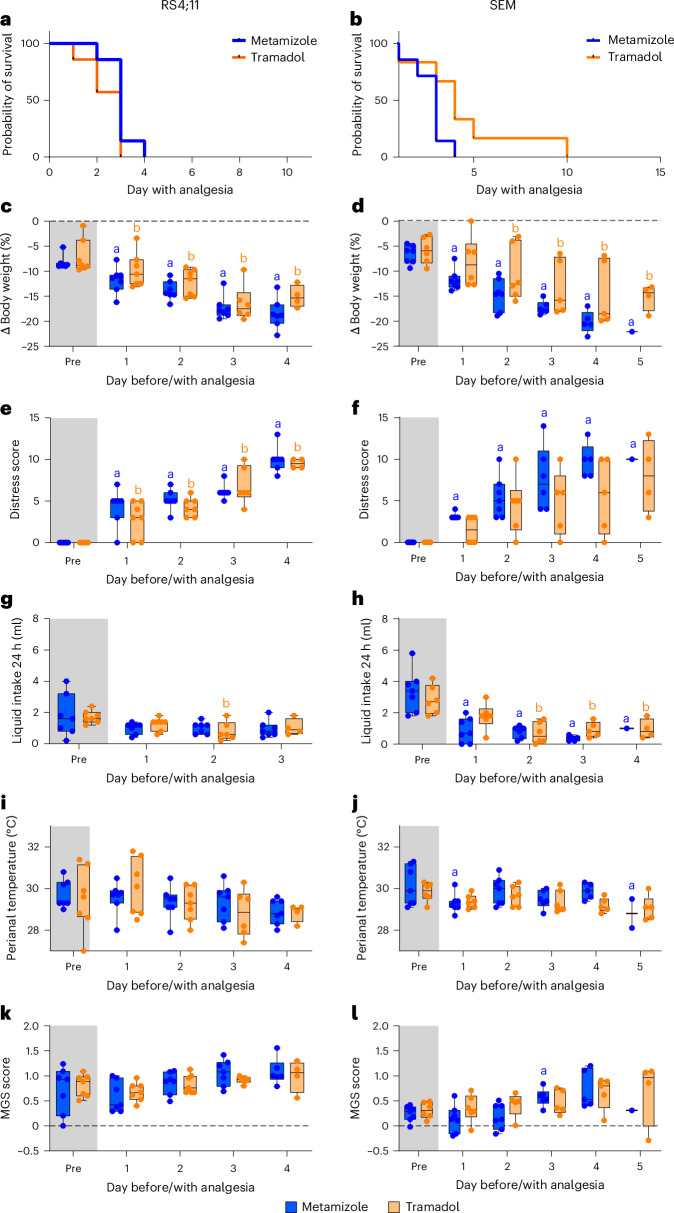
Comparison of the effects of the two oral analgesic treatments on clinical parameters in the cell-induced ALL models. **a**,**b**, Probability of survival for the RS4;11 (**a**) and SEM (**b**) models after oral treatment with either metamizole (3 mg/ml) or tramadol (1 mg/ml) in the drinking water. **c**,**d**, Body weight change in the RS4;11 (**c**) and SEM (**d**) models. **e**,**f**, Distress score in the RS4;11 (**e**) and SEM (**f**) models. Body weight change and distress scores were quantified during the last regular assessment without analgesia (pre) and on the first days (1–5) with analgesic treatment of either metamizole or tramadol. **g**,**h**, Liquid intake the RS4;11 (**g**) and SEM (**h**) models. **i**,**j**, Perianal temperature in the RS4;11 (**i**) and SEM (**j**) models. **k**,**l**, MGS in the RS4;11 (**k**) and SEM (**l**) models. Liquid intake, perianal temperature and MGS were quantified at the late stage of ALL progression, without analgesia (pre) and on the first days (1–5) with analgesic intervention. Statistics (**c**–**l**): mixed-effects model with Šidák multiple-comparison test. Significant differences (*P* < 0.05) to pre shown for metamizole (‘a’) and tramadol (‘b’) are shown. Data are presented as box plots showing the 25th–75th percentiles, with minimum and maximum values. In addition, each data point represents an individual animal at a specific time point. Detailed information about statistical results is listed in Supplementary Table 2. RS4;11: metamizole *n* = 7 (3 males, 4 females); tramadol *n* = 7 (4 males, 3 females); SEM: metamizole *n* = 7 (3 males, 4 females), tramadol *n* = 6 (3 males, 3 females). Source data

When severe leukemia progression and pain-related criteria such as body weight loss >10%, blast frequency in the blood >20%, squeaking due to pain or abnormal posture were recognized during leukemia progression, the mice were randomly assigned to the two analgesic groups, and welfare assessment was performed daily until euthanasia of the mice. For the RS4;11-injected mice, a median survival of 3 days was observed with metamizole or tramadol treatment (Fig. 2a). For mice with SEM cells, a median survival of 3 days and 4 days was observed for metamizole treatment and tramadol administration, respectively (Fig. 2b). Directly after administration of both analgesic components, a significant reduction in body weight compared with the last day without analgesia was noticed for the RS4;11 cell-injected mice (metamizole: day (d)1 *P* = 0.0011, d2–4 *P* < 0.0001; tramadol: d1 *P* = 0.0135, d2–4 *P* < 0.0001, mixed-effects analysis; Fig. 2c). Mice injected with the SEM cells showed a significant reduction in body weight on all days during metamizole treatment (d1 *P* = 0.0004, d2–5 *P* < 0.0001, mixed-effects analysis), while the body weight after tramadol administration was significantly reduced after the second day until the fifth day of application (d2 *P* = 0.0030, d3–5 *P* < 0.0001, mixed-effects analysis; Fig. 2d). A significant increase in the distress score was noticed throughout the administration of both analgesics during the late progression of RS4;11 cells (metamizole: d1 *P* = 0.0087, d2 *P* = 0.0001, d3-4 *P* < 0.0001; tramadol: d1 *P* = 0.0461, d2 *P* = 0.0002, d3-4 *P* = 0.0022; mixed-effects analysis; Fig. 2e). For the SEM cell-injected mice, a significant increase in distress score was quantified in the metamizole-treated mice only (metamizole: d1 *P* < 0.0001, d2 *P* = 0.0061, d3 *P* = 0.0200, d4 *P* = 0.0016; mixed-effects analysis; Fig. 2f). The daily liquid intake was significantly reduced on the second day of tramadol administration in the RS4;11 cell-injected mice (d2 *P* = 0.0443, mixed-effects analysis; Fig. 2g). By contrast, SEM cell-injected mice showed a significant reduction in liquid intake with both analgesics (metamizole: d1–3 *P* < 0.001, d4 *P* = 0.0274; tramadol: d2 *P* = 0.0002, d3 *P* = 0.0013, d4 *P* = 0.0030; mixed-effects analysis; Fig. 2h). No significant changes in perianal temperature were seen with both analgesics in the RS4;11 model (Fig. 2i), while metamizole led to a significant reduction of perianal temperature only on the first and fifth day of treatment in the SEM cell-injected mice (d1 *P* = 0.0125, d5 *P* = 0.0129, mixed-effects analysis; Fig. 2j). No significant changes in the MGS score were noticed for the RS4;11 cell-injected mice (Fig. 2i), while a significant increase of the MGS score was noticed for the SEM cell-injected mice on the third day of metamizole treatment only (d3 *P* = 0.0148, mixed-effects model; Fig. 2l).

**Fig. 3:**
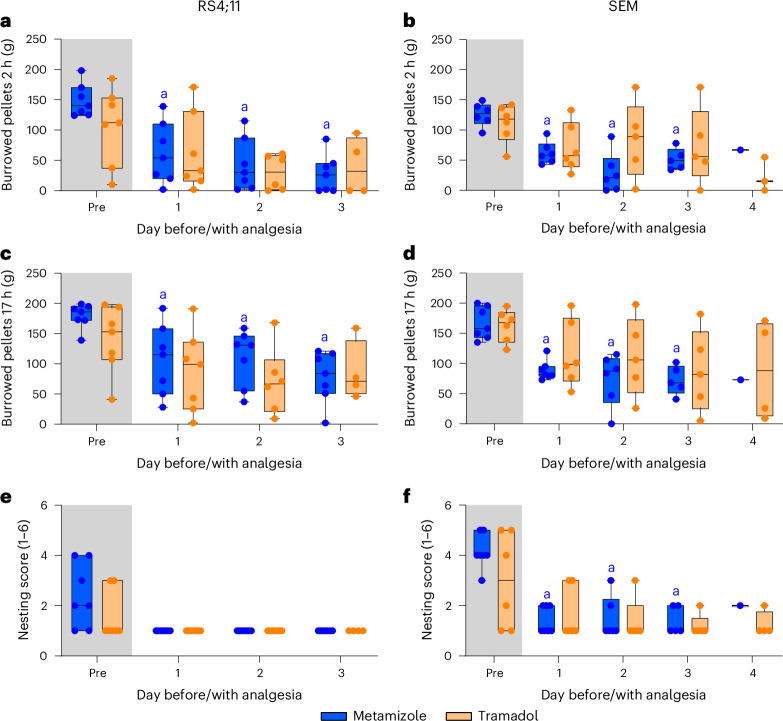
Comparison of the effects of the two oral analgesic treatments on behavioral parameters in the cell-induced ALL models. **a**–**d**, Burrowing activity for 2 h (**a** and **b**) and 17 h (**c** and **d**) in the RS4;11 (**a,** **c**) and SEM (**b** and **d**) models. **e**,**f**, Nest building for the RS4; 11 (**e**) and SEM (**f**) models. Burrowing activity and nest building were quantified during the last regular assessment without analgesia (pre) and on the first days (1–4) of analgesic treatment with either metamizole (3 mg/ml) or tramadol (1 mg/ml). Statistics: mixed-effects model with Šidák multiple-comparison test. Significant differences (*P* < 0.05) to pre shown for metamizole (‘a’) and tramadol (‘b’). Data are presented as box plots showing the 25th–75th percentiles, with minimum and maximum values. In addition, each data point represents an individual animal at the specific time point. Detailed information about statistical results is listed in Supplementary Table 3. RS4;11: metamizole *n* = 7 (3 males, 4 females); tramadol *n* = 7 (4 males, 3 females); SEM: metamizole *n* = 7 (3 males, 4 females), tramadol *n* = 6 (3 males, 3 females). Source data

Compared with the last assessment without analgesia, the total amount of burrowed pellets after 2 h and 17 h was significantly reduced after metamizole administration, but not for tramadol-treated mice, in both ALL models until the third day of administration (RS4;11, B2h: d1 *P* = 0.0012, d2 *P* = 0.0002, d3 *P* = 0.0029; RS4;11, B17h: d1 *P* = 0.0241, d2 *P* = 0.0020; d3 *P* = 0.0002; SEM, B2h: d1 *P* = 0.0028; d2 *P* = 0.0039, d3 *P* = 0.0068; SEM, B17h: d1 *P* = 0.0036, d2 *P* = 0.0202, d3 *P* = 0.0015; mixed-effects model; Fig. 3a–d). No significant differences in the nesting score were quantified for both analgesics in the two xenograft models (Fig. 3e,f).

### Data-based comparison of analgesic treatment with the RELSA algorithm

**Fig. 4:**
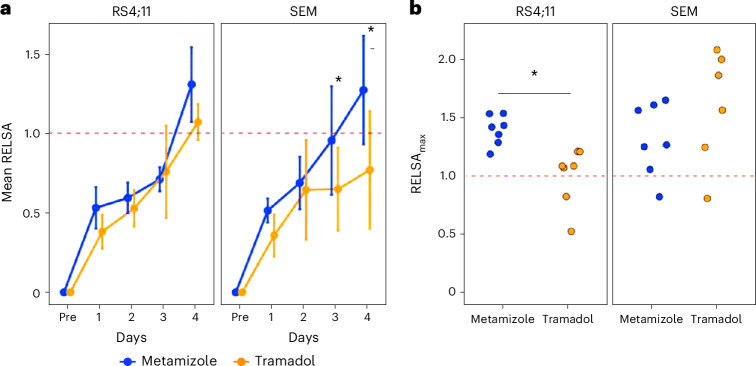
Comparison of the effects of the two oral analgesic treatments on RELSA algorithm. **a**, The mean RELSA was calculated for the last progression values without analgesia (pre) and on the following days (1–4) with analgesic treatment by administration of either metamizole or tramadol in the RS4;11 or SEM models. Statistics: ANOVA with post-hoc test revealed significant differences (*P < 0.05) for the SEM cell line after 3 and 4 days of analgesia between the metamizole and tramadol group (day 3: *Ȓ* = 0.3096, d.f. 40.9, *P = 0.0317; day 4: *Ȓ* = 0.5290, d.f. 42.7, *P = 0.0007). **b**, The RELSA_max_ was analyzed separately for mice treated with tramadol or metamizole in the SEM or RS4;11 model. Statistics: *t*-test, significant difference (*P < 0.05) was seen for the RS4;11 cell line (*t* = 3.7403, d.f. 12, *P = 0.002821). Data are presented as medians with 95% confidence intervals. RS4;11: metamizole *n* = 7 (3 males, 4 females); tramadol *n* = 7 (4 males, 3 females); SEM: metamizole *n* = 7 (3 males, 4 females), tramadol *n* = 6 (3 males, 3 females). More details about statistical analysis are listed in Supplementary Table 4. Source data

To evaluate the overall analgesic effects of metamizole or tramadol during leukemia progression in both ALL xenograft models, we used the RELSA algorithm. The RELSA was calculated using the five most informative input variables: body weight change, nesting score, distress score, liquid intake and MGS score. The RELSA was calculated for the last regular day of assessment during ALL progression (pre) before analgesic treatment and the first four days (1–4) of adding metamizole or tramadol to the drinking water. For the RS4;11-derived ALL model, no significant difference was determined by the RELSA for the two analgesics. However, a significantly higher mean RELSA was seen in the SEM-induced model for metamizole on days 3 and 4 after starting analgesic treatment when compared with tramadol (d3 *P* = 0.0317; d4 *P* = 0.0007, ANOVA; Fig. 4a). In addition, the RELSA_max_ was calculated for the phase of continuous analgesic treatment. A significantly higher RELSA_max_ was observed for the metamizole-treated mice compared with the tramadol-treated mice in the RS4;11-model (*P* = 0.0028, *t*-test). Nevertheless, no significant difference in the RELSA_max_ values was seen between the metamizole-treated mice and the tramadol-treated mice in the SEM cell model (Fig. 4b).

### Quantification of leukemic blast progression in both ALL xenograft models

Bioluminescence imaging and measurement of blast frequency in the blood were used to quantify the proliferation of leukemic blasts. No significant differences in leukemic blast progression were seen for the mice before assignment into the two different analgesic groups for both ALL models (SEM; RS4;11; Supplementary Fig. 3).

For the data collected at the individual endpoint of each mouse, no significant differences were seen between metamizole or tramadol treatment for the blast frequency in the blood, bone marrow or spleen in both cell line-induced ALL-xenograft models.In addition, no significant differences in spleen weight or size were observed between metamizole- and tramadol-treated mice in either cell line-induced ALL xenograft model (Supplementary Fig. 4).

## Discussion

### Summary of results

The present study evaluated the effectiveness of two different on-demand analgesics in the murine ALL-xenograft model induced by injection of two distinct cell lines. Physiological and behavioral parameters such as perianal temperature, burrowing behavior and nesting activity indicated a greater impairment of health in metamizole-treated mice compared with those treated with tramadol. Multifactorial data analysis revealed improved animal welfare under tramadol treatment, demonstrated by significantly lower mean RELSA with tramadol treatment in the SEM-induced model and lower RELSA_max_ in the RS4;11-derived model compared with metamizole treatment.

### Tramadol is a better analgesic compared with metamizole in ALL models

According to these results, we hypothesize that tramadol is a more effective analgesic compared with metamizole in the ALL-xenograft model. The MGS score might be one of the most important parameters in the present study to assess pain in mice^32,33^. No significant increase in the MGS score was seen in the RS4;11-injected mice for both analgesics (Fig. 2k). For the SEM cell-injected mice, a significant increase in the MGS score was detected only in the metamizole-treated mice (Fig. 2j). However, the characterization of analgesic effectiveness should not only rely on the MGS score. A clear limitation of this study that cannot be excluded is that the MGS data could be biased by the two different assessment protocols (video in box and in the home cage). For this reason, burrowing and nesting activity were additionally assessed, given that both parameters are known to indicate postsurgical^34,35^ or chemical-induced pain^36,37^. Analysis of nesting behavior indicated no significant impairment in mice after both analgesics were administered in the xenograft models. The burrowing activity for 2 and 17 h was significantly reduced in both ALL models after metamizole treatment, but not after tramadol treatment (Fig. 3). These results support the hypothesis that tramadol is the more effective analgesic for both ALL xenograft models. In other studies, opioids such as tramadol have been more effective compared with nonsteroidal anti-inflammatory drugs in murine bone cancer models^24,38^. However, a study using a mouse model for bone cancer^39^ also demonstrated the analgesic effect of metamizole. So far, no study has made a direct comparison between metamizole and tramadol administration in murine models of hematopoietic malignancies.

It is difficult to differentiate between pain resulting from insufficient analgesic dosing and the side effects arising from adequate analgesia. The basis of effective oral analgesia is the consistent drinking behavior of the mice. However, in both ALL models, a significant reduction in daily liquid intake was observed for both analgesics. The impact of providing wet food soaked with the respective analgesic on the liquid intake was not quantified. We could also not calculate the actual intake of the analgesics, because the intake of wet food was not assessed. The inability to quantify the exact amount of analgesic consumed is a clear limitation of this study. However, the range for recommended effective doses for both analgesics is very large: 150–500 mg/kg (refs. ^40,41^) for metamizole and 25–80 mg/kg (refs. ^24,42,43^) for tramadol in mice or rats. These results rely on either the bioavailability of active metabolites in the plasma or the analysis of antinociceptive effects. The comparison of different analgesics and their effects in a specific animal model might be more valid, regarding the refinement of this animal model.

The body weight loss during the analgesic treatment might be related to the taste of the specific analgesic. This hypothesis could be verified in a simultaneously running study for the analgesic metamizole, where we observed that NSG mice had problems habituating to different metamizole formulations, as indicated by significantly reduced liquid intake and body weight^44^. In addition, the sweetened formulation of metamizole led to longer significant body weight reduction in NSG compared with the nonsweetened formulation^44^. The reduced oral liquid intake might lead to significant body weight loss and affect other physiological or behavioral parameters.

In a murine model for osteotomy, the oral administration of high tramadol concentrations negatively affected mice’s well-being^20^. In the mentioned study, animals treated with low tramadol concentrations (0.01 mg/ml) recovered one day earlier from a significant body weight reduction after osteotomy compared with mice treated with high tramadol concentrations (1 mg/ml). In the present study, we quantified from the liquid intake an average tramadol dose of 51.6 mg/kg in 24 h for the RS4;11 cell-injected mice and 80.5 mg/kg for the SEM cell-injected mice. The effective dose might be actually higher, given that the analgesic intake via wet food was not quantified. However, the bioavailability in plasma and the antinociceptive effects of tramadol in mice or rats last between 2 h and 6 h (refs. ^42,45,46^). Therefore, the average tramadol intake for both ALL models is quite low, between 12.9 mg/kg and 20.1 mg/kg within 6 h. A reduction of the tramadol concentration in the drinking water would decrease the analgesic intake, especially a 10-fold reduction of tramadol concentration as described by Jirkof et al.^20^. Tang et al. reported no significant changes in body weight, nesting and burrowing behavior in healthy mice after administration of 1 mg/ml tramadol in the drinking water^18^. Another study indicated no significant reduction in body weight and water intake after application of the high tramadol concentration (1 mg/ml) in the drinking water during dextran sulfate sodium-induced colitis in mice^19^. In consideration of the above-mentioned studies, we believe that a reduction of the tramadol concentration might not be useful.

Hence, although the influence of side effects or insufficient analgesic effect of metamizole or tramadol cannot be excluded, the results of this study indicate that tramadol is a more effective analgesic than metamizole for the ALL-xenograft model. In line with our findings, the oral administration of tramadol proved to be more effective than metamizole in murine models for pancreatitis^18,47^ and colitis^19^. These results support the idea that tramadol might be generally preferred as an oral analgesic over metamizole for use in mice regardless of animal model-specific pain.

### The RELSA algorithm is a suitable tool to compare the effect of different pain management protocols

The RELSA procedure was originally established to compare the evidence-based severity of different animal models or interventions. Transmitter implantation was used as a reference dataset for qualitative severity context for moderate severity. The RELSA_max_ of 1.0 indicates the highest severity assessed in animals after transmitter implantation. For two tramadol-treated mice in the RS4;11 model, the RELSA_max_ was below 1.0. For the SEM cell-injected mice in both analgesic groups, a RELSA_max_ under 1.0 was calculated for one animal, respectively. These animals could be quantified with an evidence-based severity of moderate, while all the mice indicating a RELSA_max_ higher than 1.0 would be categorized in a higher severity level such as severe. However, because all mice were euthanized as soon as humane endpoint criteria according to the clinical score sheet (Supplementary Table 1) were met, this evidence-based categorization of the severity level was expected. In the future, the establishment of early humane endpoints, as already described in other murine cancer models^48^, might be useful to reduce the overall severity in this animal model.

An explicit limitation of the present study is the relatively small group size of six to seven mice for each analgesic group and each cell line. Following the reduction aspect of the 3R principle, an absolute minimum of animals should be used to answer a scientific question. Due to the small group size, no significant differences were observed between the analgesic groups for all welfare parameters (Figs. 2 and 3). Nevertheless, the combination of different parameters by the RELSA algorithm revealed a significantly lower severity for tramadol-treated mice, even with these small-sized groups. This suggests that RELSA cannot only be used for comparing the severity of different animal models, as published for the comparison of gastrointestinal animal models^49^, murine models for pancreatic cancer^50^ or even different mouse models for depression^51^, but also for assessing the effects of different treatments on animal well-being. This finding is in accordance with our latest report comparing the dose-dependent severity of STZ-induced diabetes in NSG mice^52^. We highly recommend using the welfare parameters and the RELSA algorithm for individual animal model-specific refinements in future experiments.

### Analgesia has no effect on the proliferation of the leukemic blasts

This study used two cell lines to induce leukemia in NSG mice: the slow-proliferating RS4;11 cells, and SEM cells showing faster engraftment. The differences between metamizole and tramadol were more evident for the fast-proliferating SEM cell line. SEM cell-injected mice were mainly euthanized due to a body weight loss >20%, while the euthanasia criterion for the RS4;11 cells was primarily a blast frequency in the blood >30%. One could hypothesize that faster engraftment and severe body weight loss are associated with painful conditions, which could facilitate the detectability of analgesic’s effectiveness. Nevertheless, RS4;11 cell-injected mice displayed a significantly higher RELSA_max_ after metamizole administration compared with tramadol administration, indicating a significantly lower severity for tramadol-treated mice and potential adverse side effects for metamizole. In conclusion, in both ALL-xenograft models, tramadol treatment improved the welfare of mice at the end of leukemia progression compared with metamizole treatment.

### On-demand, analgesic treatment should be preferred during ALL progression

Concerning the current study’s design, one could ask why tramadol or metamizole were used as on-demand analgesia and not administered continuously after injection of RS4;11 or SEM. The administration of analgesia can lead to side effects in mice. In a previous study, we observed that the administration of metamizole via drinking water led to significant body weight loss and reduced liquid intake in healthy mice of different mouse strains^44^. Considering these side effects, analgesic treatment should start when pain symptoms are recognized. During the first weeks of leukemia progression, data-based severity assessment parameters revealed minimal impact on the mice’s well-being for both cell-lines, reflecting the clinical situation in humans. As indicated by significantly reduced body weight and nesting activity, as well as a significantly increased MGS score (Supplementary Figs. 1 and 2), an impairment of well-being was observed after 7 weeks of leukemia progression for the RS4;11 cell-injected mice, and after 5 weeks for SEM cells. The MGS score significantly increased within the last 2 weeks of leukemia progression for both cell lines (Supplementary Fig. 1i,j). These results indicate that the mice might already experience pain during the last 2 weeks of leukemia progression and, therefore, suggest that body weight loss and blast frequency are inadequate criteria to determine the start of analgesic treatment. Thus, the MGS should be used as an additional criterion to determine the start of analgesia in future animal experiments to increase animal welfare.

The present study revealed no difference in cell engraftment and ALL progression for the animals assigned into the different treatment groups until the start of analgesic treatment (Supplementary Fig. 3). At the individual endpoint of each mouse, which also marks the end of the analgesic treatment, we also observed no significant difference in the leukemic engraftment between metamizole- and tramadol-treated mice (Supplementary Fig. 4). Because the analgesic treatment starts in a late stage of progression and has a duration of only 3–4 days, these results were expected. To evaluate the impact of any analgesic on leukemia progression, a control group without any on-demand analgesic would be necessary. However, the occurrence of pain at the end of leukemic progression denies ethical acceptability for this control group. Of note, it has been reported that long-term analgesic treatment with tramadol or metamizole can influence the proliferation of solid tumors, as seen for pancreatic cancer^53^, hepatic cellular carcinoma^54^ or mammary carcinoma^55^. Furthermore, analgesic treatment can reduce metastasis in cancer models^56^. Therefore, we cannot rule out a possible impact of long-term analgesic treatment on the proliferation of leukemic blasts. However, tramadol is commonly used for analgesia in the clinic to treat moderate to strong pain in patients with leukemia^57^. The use of tramadol in a preclinical xenograft model induced by human cells, therefore, might increase the translational potential of the study outcome.

## Conclusion

The results of the present study suggest that tramadol is a superior analgesic over metamizole in cell-induced ALL-xenograft models. This conclusion is supported by the RELSA analysis, which revealed a lower severity in the ALL mice under tramadol treatment.

## Methods

### Cell lines and cell culture

GFP- and enhanced firefly luciferase (ffLuc)-transduced human precursor ALL cell lines SEM and RS4;11 were provided by Prof. Irmela Jeremias (Helmholtz Center Munich, Germany). Both cell lines were stably transduced with GFP and enhanced firefly luciferase (ffLuc) in the pCDH-EF1-MCS-T2A-copGFP vector (System Bioscience) using EcoRI and BamH1. Cell transduction and lentivirus production were carried out as described by Terziyska et al.^58^. Cells were cultured in IMDM medium (SEM) or Alpha medium (RS4;11) supplemented with 10% heat-inactivated fetal calf serum (PAN-Biotech) and 1% penicillin–streptomycin (PAN-Biotech) and cultured at 37 °C and 5% CO_2_ before collection, washing in phosphate-buffered saline and tail-vein injection.

### Animals

The local authority Landesamt für Landwirtschaft, Lebensmittelsicherheit und Fischerei Mecklenburg-Vorpommern approved the animal experiments (7221.3-1-063/20-5). The experiments were performed under the German animal protection law and the European Directive 2010/63/EU^9,10^. Breeding pairs of immune-deficient NSG mice were purchased from Charles River Laboratories and bred under pathogen-free conditions in individually ventilated cages in our facility at the University Medical Center Rostock. The mice’s health state was routinely checked according to FELASA Guidelines. Over the past 2 years, *Helicobacter* sp., *Rodentibacter pneumotropicus* and murine norovirus were detected in some mice of immunocompetent mouse strains. These mice were not used for breeding or experiments. Before the experiment, all NSG mice were in good health. During the experiments, NSG mice were singly housed in type III cages (Zoonlab GmbH) with filter tops at a 12-h dark:light cycle, with a room temperature of 21 ± 2 °C, relative humidity of 60 ± 20% with food (pellets, 10 mm, ssniff-Spezialdiäten GmbH) and water with or without added analgesic ad libitum. Enrichment was provided by nesting material (shredded tissue paper, Verbandmittel GmbH), paper rolls (75 × 38 mm, H0528–151, ssniff-Spezialdiäten GmbH), as well as wooden sticks (40 × 16 × 10 mm, Abedd). The handling of the mice during the experiment was performed via cup handling and securing the tail between the fingers. The mice had at least 1 week of acclimatization in the new animal room before the experiments started.

### Animal models

NSG mice (male and female, 13–20 weeks) were anesthetized with isoflurane (induction: 3 vol.%, maintenance: 1–2 vol.%) and received an intravenous injection of human ALL precursor cells (2.5 × 10^6^, SEM-GFP-ffLuc or RS4;11-GFP-ffLuc in 100 µl phosphate-buffered saline) via tail vein. Seven male (16.8 ± 0.07 weeks; 26 ± 0.76 g body weight) and seven female NSG mice (18 weeks ± 1.6 weeks, 31.6 ± 0.83 g body weight) received the RS4;11 cells. Seven male (12.9 ± 0.14 weeks, 28.8 ± 1.4 g body weight) and seven female NSG mice (23.8 ± 1.9 weeks, 23.8 ± 1.9 g body weight) were used for the injection of SEM cells. A total of 28 NSG mice were used for the present study. For leukemic blast injection, mice were placed in a lateral position on a heating plate (37 °C), and the cells were slowly injected by using a 30 G needle (BD) and a catheter (ICU Medical). The engraftment of leukemic blasts was evaluated once a week by bioluminescence imaging with the NightOWL LB 983 Imaging System (Berthold Technologies) and Indigo software (Berthold Technologies, version 1.04). For this purpose, animals were intraperitoneally injected with 4.5 mg d-Luciferin (GOLDbiotechnology). Five minutes after injection, mice were anesthetized with isoflurane (induction: 3 vol.%, maintenance: 1–2 vol.%) and imaged in dorsal and ventral position for 60 s each side at 560 nm.

### Assessment of clinical parameters

Body weights and distress scores were assessed daily during the whole experiment. Body weight change (%) was calculated as the difference from baseline on a given day during the first week before leukemic blast injection in healthy mice. For distress score assessment, mice were observed daily in the home cage and during the handling procedure for body weight measurement to determine whether any health-limiting criteria were present (Supplementary Table 1). Single scores of all fulfilled criteria were summed up. Healthy mice had a sum score of 0. Liquid intake was measured every 24 h using polystyrene pipettes according to Bachmanov et al.^59^.

The assessment of pain behavior was performed via MGS, as described by Langford et al.^26^. The mice were transferred into separate transparent polycarbonate boxes (box: 5 × 5 × 9 cm) and in a light tent with a black background and additional light from the front. After an acclimatization time of 5 min, the mice were filmed for a further 5 min with a single-lens reflex camera (Canon EOS 70). Three pictures of each mouse were captured per time point. All images of the experiment were then blinded, randomized and scored by three independent people, by assigning a value of 0, 1 or 2 for orbital tightening, nose bulge, check bulge, ear position and whisker change. For each mouse, the MGS score was calculated according to Langford et al. as the average for each distinct time point, normalized to baseline values obtained before cell injection in healthy mice^26^. During analgesic application, three people scored the mice in their home cages to minimize the stress for the animals, which were already in poor health.

The perianal surface temperature was measured three times with a contactless infrared thermometer (Wepa Apothekenbedarf GmbH & Co. KG) for each time point, and the mean value was calculated^27^.

At the beginning of the experiment, the mice were trained two times to acclimatize to the MGS boxes and to habituate to the perianal temperature measurement. The physiological parameters MGS, liquid intake and perianal temperature were assessed before tumor cell injection in healthy mice and once a week during engraftment of the human leukemic blasts. During analgesic treatment, these parameters were assessed daily.

### Assessment of behavioral and biochemical parameters

The burrowing behavior was assessed by placing a 3D-printed tube (15 × 6.5 × 6.5 cm) filled with 200 ± 1 g food pellets (ssniff-Spezialdiäten GmbH) into the home cage 2–3 h before the dark phase^28^. The number of burrowed pellets was evaluated after 2 h and the following day (after approximately 17 h). Nesting activity was quantified by placing a cotton nestlet (ZOONLAB GmbH) into the home cage 1-2 h before dark phase. The nest appearance was evaluated the following day, according to a 1–5-point scale from Deacon et al.^29^. In addition, a score of 6 was assigned to a perfect nest, characterized by a crater-like structure with more than 90% of the nest wall higher than the mouse.

For FCM, feces were collected once a week, 24 h after cage change during leukemia progression. The feces were dried for 4 h at 65 °C and stored at −4 °C. To quantify FCM, 50 mg of pulverized feces was extracted in 1 ml methanol (80%) for analysis using a 5α-pregnane-3β,11β,21-triol-20-one enzyme immunoassay^60^.

### Analgesic administration

As soon as health-related criteria, such as body weight loss >10% or blast frequency in the blood >20%, were recognized during leukemia progression, the mice, with consideration of sex, were randomized into the analgesic groups for metamizole or tramadol. The other two criteria listed on the score sheet to start the analgesic treatment, that is, squeaking due to pain during handling, or abnormal posture, were not observed during this experiment. The concentration of metamizole (3 mg/ml) was chosen so that, with an average metamizole intake of healthy mice (3–5 ml per 24 h), the recommended doses from 150–500 mg/kg were achieved within 6–8 h (refs. ^61–65^). The concentration of tramadol (1 mg/ml) was used to achieve the recommended concentrations of tramadol 25–80 mg/kg (refs. ^24,42,43^) within 2–6 h (refs. ^42,45,46^). In the RS4;11 model, three male and four female mice received metamizole, while four male and three female mice were assigned to the tramadol treatment group. For the SEM cell-injected mice, three males and four females were treated with metamizole, while three males and three females received tramadol. One male mouse in the SEM cell-induced model did not receive any analgesia because the mouse had to be euthanized immediately due to abnormal respiratory breathing. One day before euthanasia, no criteria for analgesia application were recognized in the specific mouse. The sample size of seven mice for each cell line and analgesic was calculated when planning the experiment with a significance level of 0.05, a power of 0.8 and Cohen’s *d* of 1.4995. Metamizole (3 mg/ml) and tramadol (1 mg/ml) were administered via drinking water with daily renewal. Wet food soaked with the respective analgesic was also applied daily to prevent dehydration and fast body weight loss.

The mice received analgesia until humane endpoint criteria such as 20% bodyweight loss, abnormal breathing and apathy (restricted movement) or blast frequency in the blood >30% were met (Supplementary Table 1). The maximal tumor burden permitted by the ethics committee was defined as a blast frequency in the blood exceeding 30%. As soon as more than 30% blast in the blood was quantified, or one of the other humane endpoint criteria (Supplementary Table 1) was met, the mice were euthanized immediately. No control group without analgesia was applied because administration of analgesia is legally required as soon as signs of pain are recognized. The researchers were not blinded to the different analgesic treatment groups, as blinding was impossible due to the yellow coloration of metamizole in the water. This study is not preregistered.

### Reference data for the RELSA algorithm from transmitter implantation in mice

The data from mice after transmitter implantation were used as reference data for the RELSA algorithm. ETA-F10 telemeters (Data Science International) were implanted into the abdominal cavity of ten BALB/cANCrl mice (four females, six males; 16–20 weeks). This animal experiment was also approved by the authority Landesamt für Landwirtschaft, Lebensmittelsicherheit und Fischerei Mecklenburg-Vorpommern (7221.3-1-068/21). These mice were anesthetized by inhalation of isoflurane (induction: 4 vol.%, maintenance: 1–2 vol.%). Continuous analgesia was applied by metamizole (3 mg/ml) via the drinking water, and carprofen (5 mg/kg, Rimadyl, Pfitzer GmbH) was subcutaneously injected once directly before surgery. The abdomen and the thorax of the mice were shaved, a midline laparotomy was performed and the telemetric device was placed into the abdomen of the mouse. The negative electrode was sutured into the Musculus pectoralis major, and the positive electrode was sutured on the Musculus external oblique. The peritoneum was closed with coated 5–0 vicryl sutures (Johnson & Johnson Medical GmbH), and the skin was closed with 5–0 prolene suture (Johnson & Johnson Medical GmbH) by a single-knots seam. The distress parameters were evaluated for these mice before the surgical procedure (days −6, −5 and −4), directly after surgery (day 0) and on the following recovery days (days 1, 2, 3, 7, 15, 22 and 24).

### Data analysis

The data for Figs. 2–4 and Supplementary Figs. 1–4 were analyzed and graphed with GraphPad Prism 8.4.3 (GraphPad Software). In Supplementary Figs. 1 and 2, the data are shown as box plots, indicating upper and lower quartiles, single values, and median and min–max values as whiskers. Statistical analysis was performed using repeated-measures one-way ANOVA followed by Dunnett’s multiple-comparison test, or alternatively by a mixed-effects model and the Friedman test. In Figs. 2 and 3, the data are shown as box plots, indicating upper and lower quartiles, single values, and median and min–max values as whiskers. Statistical analyses included repeated-measure mixed-effects analysis with Geisser–Greenhouse correction for sphericity control, followed by Šidák’s multiple-comparison test. Differences from the prevalues, defined as the last regularly assessed value at the late stage of tumor progression for each parameter, were considered significant at *P* < 0.05. For Fig. 4, the data were analyzed with the RELSA algorithm^31^ using the RELSA R package (https://talbotsr.com/RELSA/index.html, accessed on 20 November 2024). The RELSA was calculated on the basis of five variables (body weight, nesting, distress score, liquid intake and MGS). These variables were mapped against the reference data from telemeter implantation in BALB/c mice, where the same variables were assessed. The RELSA score was originally established to compare animal models and their specific severity category. Therefore, the RELSA algorithm requires a reference set of data as qualitative severity context. We used the dataset for transmitter implantation, because this surgical procedure is categorized as moderate. At each observed time point (*t*), differences to the normalized baseline in each contributing outcome measure (*i*) were calculated. For the quantitative severity context, the differences were divided by the normalized maximum-reached differences in the respective variables of the references dataset, to yield the RELSA weights (*R*_w_).

$${R}_{{\rm{w}},i}(t)=\frac{\left(\left|\begin{array}{c}100-i\end{array}\right|\right)}{\left(\left|100-{\rm{max}}_{i,{{{ref}}}}\right|\right)}.$$

Larger differences of variables were given more weight. The final RELSA score was calculated by the root mean square of the variable *R*_w_ divided by the number of variables (*N*). Input variables that contribute the least to the *R*_w_ can be excluded directly excluded from the input variables.

$${\rm{RELSA}}\left({{t}}\right)=\sqrt{\frac{{\sum }_{1}^{i}{{R}_{{\rm{w}},i}}^2}{N}}.$$

The RELSA_max_ of 1.0 represents the maximum severity of an animal in the reference dataset^31^. For Fig. 4a, the mean RELSA was calculated separately for each cell line at the late stage of ALL before the application of analgesia (pre) and on the following days with analgesia (d1–4). Each treatment group’s time-dependent progression of cell-type-specific severity was quantified using the RELSA metric. This progression was then evaluated via ANOVA, incorporating a cell-type-by-time interaction term.

Post-hoc comparisons of RELSA values between analgesia groups were also performed for each experimental day. Data from all analgesic treatment time points (d0–10) were used to calculate the RELSA_max_, representing the highest level of distress experienced by each animal from treatment initiation until euthanasia.

In Supplementary Fig. 3, data for bioluminescence signal and blast frequency are shown as mean curves and single points for each mouse. In Supplementary Fig. 4, leukemic blast engraftment at the individual endpoint is shown in bar plots depicting the mean and standard deviation. Detailed statistical analyses for each figure are provided in Supplementary Tables 2–8. Raw data are included as Source Data and Supplementary Data.

### Reporting summary

Further information on research design is available in the Nature Portfolio Reporting Summary linked to this article.

## Online content

Any methods, additional references, Nature Portfolio reporting summaries, source data, extended data, supplementary information, acknowledgements, peer review information; details of author contributions and competing interests; and statements of data and code availability are available at 10.1038/s41684-025-01615-3.

## Supplementary information

Supplementary Information Supplementary Figs. 1–4 and Tables 1–8.

Reporting Summary

Supplementary Data Source data for Supplementary Figs. 1–4.

## Source data

Source Data Figs. 2–4 Statistical source data.

## Data Availability

The data of the present study are available in the Supplementary Information. Additional information is available on request from the corresponding author. Source data are provided with this paper.
