# Supplementary material for: Improving pain management for murine orthotopic xenograft models of acute lymphoblastic leukemia
Source: Lab Anim (NY). 2025 Oct 8;54(11):313–20. doi: 10.1038/s41684-025-01615-3 (PMC12575361; doi:10.1038/s41684-025-01615-3)
Supplement: Supplementary file 1 — Supplementary Figs. 1–4 and Tables 1–8. [file 41684_2025_1615_MOESM1_ESM.pdf]

lab animal

https://doi.org/10.1038/s41684-025-01615-3

Improving pain management for murine orthotopic xenograft models of acute lymphoblastic leukemia

In the format provided by the authors and unedited

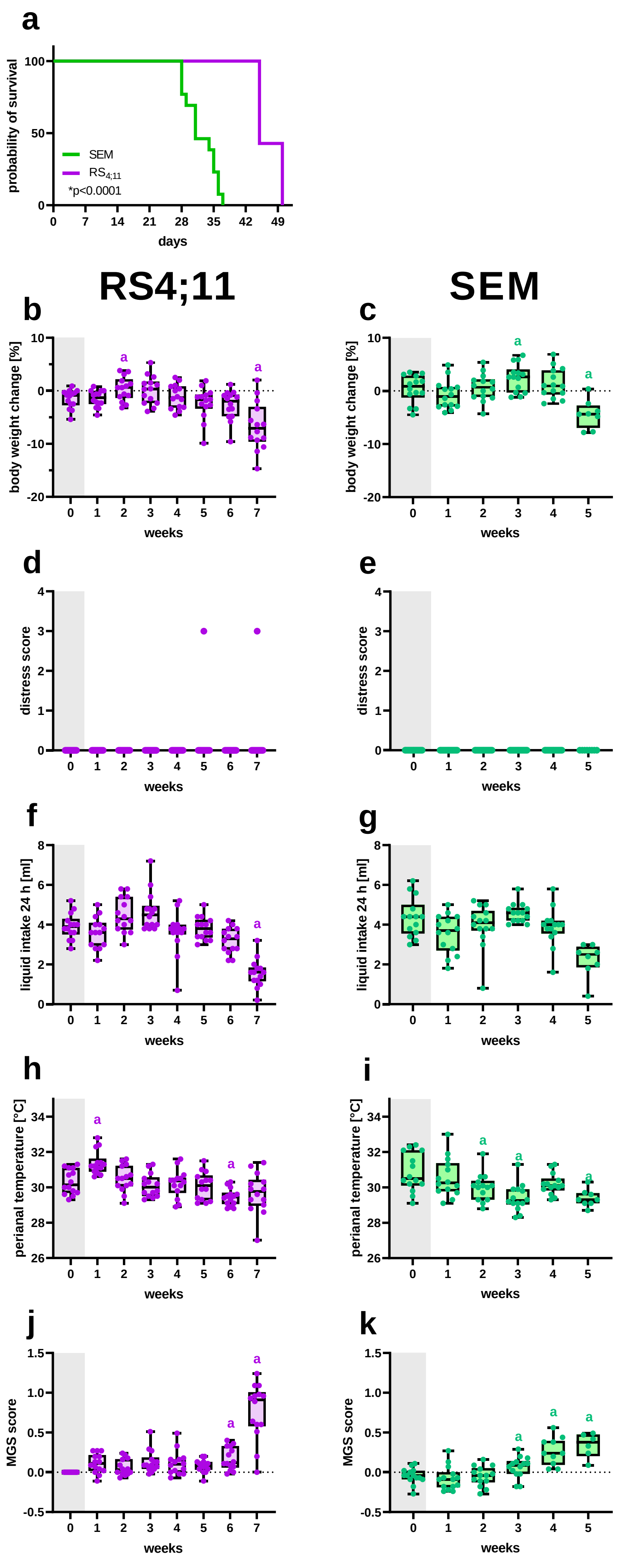

SFig1. Survival and distress of mice during leukemia progression, without analgesic treatment quantified by clinical parameters. Survival of mice after injection of human ALL precursor cell lines SEM and RS4;11 (**a**). The parameters body weight change (**b, c**), distress score (**d, e**), liquid intake (**f, g**), perianal temperature (**h, i**) and mouse grimace scale (**j, k**) were assessed one week before tumor cell injection (0) and during the following weeks after injection of RS4;11 (b, d, f, h) and SEM cells (c, e, g, i). Statistics: Log-rank (Mantel-Cox) test (a), Mixed effects model (c, e, i, k), Friedman test, followed by Dunn's test for multiple comparisons (g, f) or repeated measures one-way ANOVA and Dunnett's multiple comparisons (b, d, h, j). *P*<0.05 was considered significant. a: significant differences to week 0 (highlighted in grey). RS4;11: N=14 (7 male, 7 female); SEM: N=14 (7 male/ 7 female). Data are presented as box plot, 25-75% quartiles, min, and max as percentiles, as well as indicating single values. Details of statistical analysis are indicated in Supplementary Tab. 5.

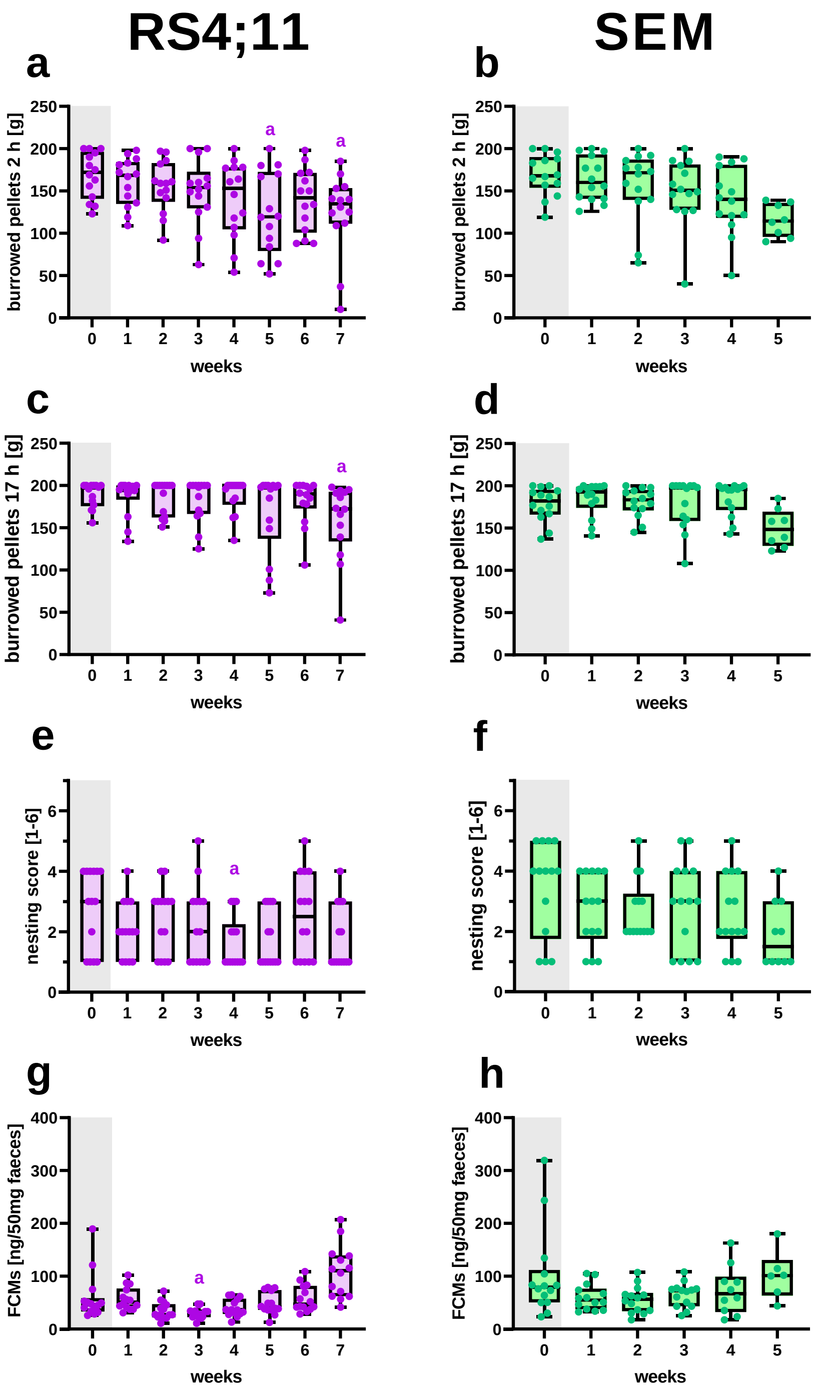

SFig.2 Impairment of animal welfare during leukemia progression without analgesic treatment quantified by behavioral, hormonal parameters. Burrowing behavior after 2 hours (**a-b**) and 17 hours (**c-d**), nesting activity (**e-f**) and fecal corticosterone metabolites (**g-h)** were quantified on the week before (0) and after injection of RS4;11 (a, c, e, g, i) and SEM cells (b, d, f, h, j). Statistics: Repeated measures one-way ANOVA followed by Dunnett's multiple comparisons (a); Friedman test, followed by Dunn's test for multiple comparisons (b-h). $P<0.05$ was considered significant. a: Significant differences to week 0 (highlights in grey). RS4;11: N=14 (7 male/ 7 female); SEM: N=14 (7 male/ 7 female) Data are presented as box plot, 25-75% quartiles, min, and max as percentiles, as well as single values. Details of statistical analysis are listed in Supplementary Tab. 6.

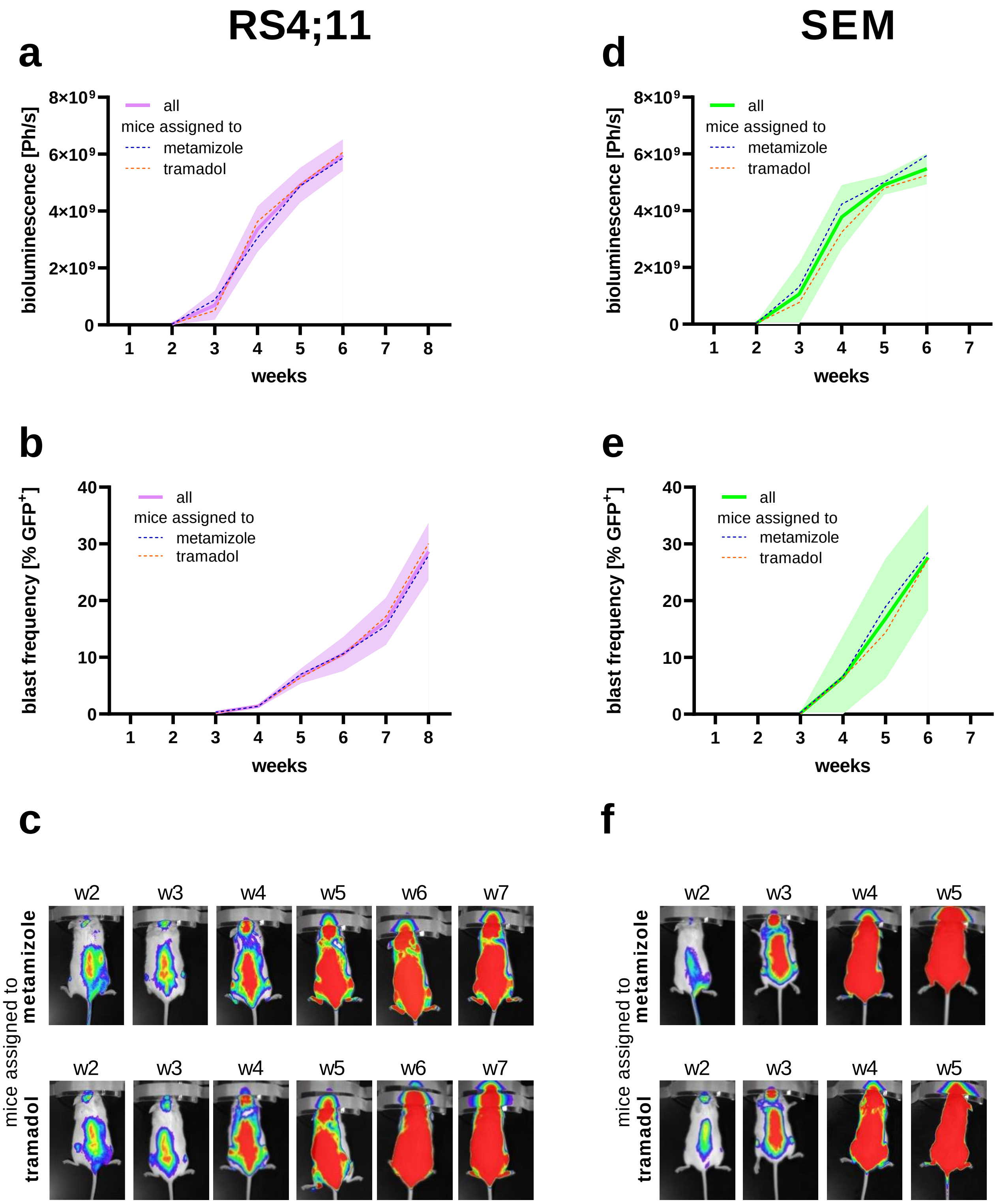

SFig.3 Progression of ALL in mice induced by human leukemic blasts (RS4;11, SEM), before administration of analgesics. Progression of leukemia either in RS4;11 (**a-c**) or SEM-induced (**d-f**) animal model is shown for all animals and the distinct subpopulations that received either metamizole or tramadol at a later stage. The engraftment of leukemic blasts was quantified once a week by bioluminescence imaging (a, d) and blast frequency in the blood (b, e). Exemplary pictures indicate the detected bioluminescence signal for the mice before assignment either to the metamizole, or tramadol treatment group (c, f), separately for each ALL-model. Data are presented as line graph with mean values (bold line) and standard deviation (shaded area), dashed lines indicate mean values of mice later assigned either to tramadol or metamizole treatment. RS4;11: N=14 (7 male/ 7 female); SEM: N=14 (7 male/ 7 female). Detailed information about the statistic is listed in Supplementary Tab. 7.

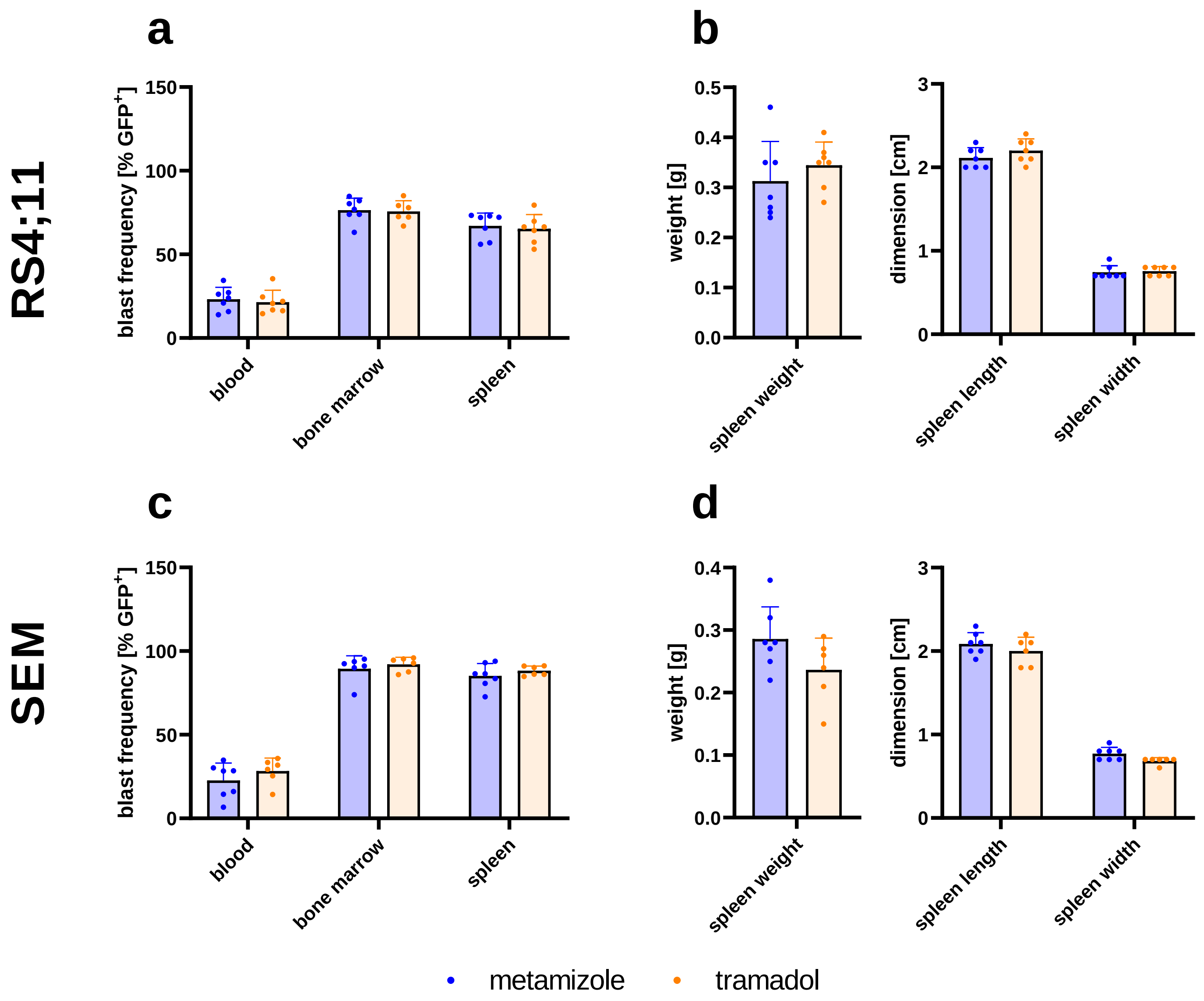

SFig.4 Final state of leukemia progression at the individual humane endpoint after metamizole or tramadol treatment. For the RS4;11 model, the final blast frequency in the blood, bone marrow, and spleen (a) and spleen weight and size (b) were evaluated for each mouse at the individual endpoint. In accordance, for mice of the SEM model, the blast frequencies in the blood, bone marrow and spleen (c) were quantified and spleen weight and size (d) were measured, separately for tramadol and metamizole treated mice. RS4;11: metamizole N=7 (3 male/ 4 female), tramadol N=6-7 (4 male/ 3 female); SEM: metamizole N=7 (3 male/ 4 female), tramadol N=6 (3 male/ 3 female). Data are presented as bar chart, error bars indicating mean ± SD, and single values are depicted. Detailed information about statistical analysis is indicated in Supplementary Tab. 8.

Supplementary Table 1. Clinical score sheet

Criteria for analgesia start	
body weight decreased > 10% (compared to initial weight)<br>blast frequency in the blood > 20%<br>squeaking due to pain during handling<br>abnormal or bent posture	

Observations

I Body weight	Score
I-a decreased > 10% (compared to initial weight)	3
I-b decreased > 20% (compared to initial weight)	4

II General condition	
appearance	1 (A)
II-a tooth displacement, too long teeth	2
II-b fur dull, ruffled or untended	2
II-c eyes unclear or squinted	3
II-d untended orifices of the body	3
II-e abnormal posture	3
II-f dehydration impairments/abnormalities	3
II-g short spasms or temporary paralysis symptoms	4
II-h persistent (>30') cramping or paralysis	4
II-i abnormal respiratory sounds or breathing, animal feels cold	4
II-j state of cachexia (body condition score: BC 2)	

III Spontaneous behavior	
III-a animal is passive or overactive	2
III-b pronounced apathy (restricted movement), hyperkinesia, isolation	4
III-c squeaking due to pain	4
III-d self-mutilation	4

IV Flight behavior after contact	
IV-a animal is moderately passive or overactive	2
IV-b distinct apathy or hyperkinesia	4

V ALL-model specific criteria	
V-a blast frequency in the blood >30%	4

Score points are stated per line as soon as one criteria applies. Even with several positive results per line, there is no addition of the points per line.

Procedures

Single score	Total score	Distress level	Measures
A		mild	Shorten teeth.
B		moderate	Inform the person in charge of the experiment. If necessary, anesthetize the animal and close the wound. Document it.
1		mild	Inform the person in charge of the experiment. A sufficient frequency of observation is necessary, consider treatment options and document it.
2-3		moderate	Inform the person in charge of the experiment. Daily observation of the animal is necessary, consider treatment options and document it.
4		severe	In agreement with the person in charge euthanasia (preferably painless after anesthesia) has to be performed. Document it.
	3-4	mild	Inform the person in charge of the experiment. Daily observation of the animal is necessary, consider treatment options and document it.
	5-10	moderate	Inform the person in charge of the experiment. Euthanasia or treating the animal plus daily observation of the animal is necessary. Document it.
	> 11	severe	In agreement with the person in charge euthanasia (preferably painless after anesthesia) has to be performed. Document it.

Supplementary Table 2. Statistics for data of figure 2

Figure	Statistic model	Effects	F (DFn, DFd)	p-value
1a	Log-rank test		X^2= 2.066	0.1506
1b	Log-rank test		X^2= 3.167	0.0751
1c	mixed-effects analysis	time	F (4, 44)= 70.40	<0.001*
		analgesic	F (1, 12) = 2.009	0.1818
		time x analgesic	F (4, 44) = 0.4808	0.7496
1d	mixed-effects analysis	time	F (5, 42) = 40.10	<0.0001*
		analgesic	F (1, 11) = 5.386	0.0405*
		time x analgesic	F (5, 42) = 1.759	0.1423
1e	mixed-effects analysis	time	F (2.659, 29.25) = 94.98	<0.0001*
		analgesic	F (1, 12) = 0.6696	0.4291
		time x analgesic	F (4, 44) = 1.260	0.3000
1f	mixed-effects analysis	time	F (2.5999, 21.83) = 23.14	<0.0001*
		analgesic	F (1, 11) = 3.722	0.0799
		time x analgesic	F (5, 42) = 1.551	0.1950
1g	mixed-effects analysis	time	F (1.838, 19.60) = 5.926	0.0111*
		analgesic	F (1,12) = 2.777	0.9959
		time x analgesic	F (3, 32) =0.4484	0.7201
1h	mixed-effects analysis	time	F (2, 32) = 20.34	<0.0001*
		analgesic	F (1, 11) = 0.3454	0.5686
		time x analgesic	F (4, 32) = 1.620	0.1933
1i	mixed-effects analysis	time	F (4, 44) = 3.523	0.0140*
		analgesic	F (1,12) = 0.02027	0.8892
		time x analgesic	F (4, 44) = 0.999	0.4178
1j	mixed-effects analysis	time	F (5, 45) = 4.590	0.0018*
		analgesic	F (1, 11) = 0.5826	0.4614
		time x analgesic	F (5, 45) = 0.8898	0.4959
1k	mixed-effects analysis	time	F (2.091, 22.48) = 6.75	0.0046*
		analgesic	F (1, 12) = 0.03140	0.8623
		time x analgesic	F (4, 43) = 0.7916	0.5371
1l	mixed-effects analysis	time	F (2.02, 16,57) = 6.965	0.0063*
		analgesic	F (1, 11) = 0.8862)	0.3667
		time x analgesic	F (5, 41) = 1.108	0.3712

Supplementary Table 3. Statistics for data of figure 3

Figure	Statistic model	Effects	F (DFn, DFd)	p-value
4a	mixed-effects analysis	time	F ((2.760; 29.44) = 14.47	<0.001*
		analgesic	F (1, 12) = 0.4857	0.4491
		time x analgesic	F (3, 32) = 0.9500	0.4281
4b	mixed-effects analysis	time	F (1.099, 7.968) = 11.85	0.0080*
		analgesic	F (1, 10) = 0.005349	0.9431
		time x analgesic	F (4, 29) = 3.368	0.0221*
4c	mixed-effects analysis	time	F (2.444, 26.07) = 12.26	<0.0001*
		analgesic	F (1, 12) = 1.261	0.2835
		time x analgesic	F (3, 32) = 0.9931	0.4221
4d	mixed-effects analysis	time	F (1.534; 11.89) = 16.18	0.0007*
		analgesic	F (1, 11)= 0.6485	0.4377
		time x analgesic	F (4, 31) = 0.6845	0.6081
4e	mixed-effects analysis	time	F (1.000, 14.67) = 9.663	0.0074*
		analgesic	F (1, 44) = 1.645	0.2064
		time x analgesic	F (3, 44) = 1.775	0.1659
4f	mixed-effects analysis	time	F (1.564; 12.12) = 21.10	0.0002*
		analgesic	F (1, 11) = 0.6686	0.4309
		time x analgesic	F (4. 31) = 1.466	0.2363

Supplementary Table 4. Statistics for data of figure 4

Figure	Statistic	Effects	SS	df	MS	DFn	p-value
5a (RS4,11)	Typ III ANOVA	time	9.0032	4	2.25079	F (44.256, 122.0686)	<0.0001*
		analgesic	0.0364	1	0.03638	F (11.656, 1.9732)	0.1862
		time x analgesic	0.1238	4	0.03095	F (44.256, 1.6785)	0.1719
5a SEM	Typ III ANOVA	time	7.1659	4	1.79147	F (40.022, 47.8363)	<0.0001*
		analgesic	0.2089	1	0.20888	F (11.340, 5.5774)	0.03701*
		time x analgesic	0.4976	4	0.12441	F(40.022, 3.3220)	0.01931*
5b	t-Test	RS4,11		12		t= 3.7403	0.002821*
	t-Test	SEM		11		t= -1.2334	0.2431

Supplementary Table 5. Statistics for data for figure S1

Figure	Statistic model	Effects	Df, F (DFn, DFd)	p-value
S1a	Log-rank test		X^2=3047	<0.0001*
S1b	RM one Way ANOVA	time	df= 7, F (3.144, 40.87) = 15.53	<0.0001*
		individual	df = 13, F (13, 91) = 6.942	<0.0001*
		residual	df = 91	
S1c	mixed-effects analysis	time	F (3.115, 36.76)	<0.0001*
S1f	Friedmann test		FS = 59.95	<0.0001*
S1h	RM one Way ANOVA	time	df= 7, F (3.613, 46.96) = 13.54	<0.0001*
		individual	df = 13, F (13, 91) = 6.448	<0.0001*
		residual	df= 91	
S1i	mixed-effects analysis	time	F (3.198, 37.74) = 8.222	0.0002*
S2j	Friedman test	time	FS= 41.58	<0.0001*
S2k	Mixed effect analysis	time	F (3.598, 39.57) = 28.76	<0.0001*

Supplementary Table 6. Statistics for data of figure S2

Figure	Statistic model	Effects	Friedman stat. FS / Df, F (DFn, DFd)	p-value
S2a	Friedman test	time	FS= 25.58	0.0009*
S2c	Friedman test	time	FS= 25.24	0.0007*
S2e	Friedman test	time	FS= 18.99	0.0082*
S2g	Friedman test	time	FS= 45.14	<0.0001*

Supplementary Table 7. Statistics for data of figure S3

Figure	Statistic model	Effects	F (DFn, DFd)	p-value
S3a	mixed-effects analysis	time	F (4, 47) = 309.6	<0.0001*
		analgesic	F (1, 12) = 0.3989	0.5395
		time x analgesic	F (4,47) = 1.312	0.2793
S3b	mixed-effects analysis	time	F (5,52) = 154.0	<0.0001*
		analgesic	F (1, 12) = 0.3030	0.5921
		time x analgesic	F (5,52) = 0.4718	0.7956
S3d	mixed-effects analysis	time	F (1.749; 29.73) = 130.0	<0.0001*
		analgesic	F (4, 23) = 1.215	0.3316
		time x analgesic	F (16, 68) = 0.5314	0.9210
S3e	mixed-effects analysis	time	F (3, 24) = 23.91	<0.0001*
		analgesic	F (1, 11) = 0.2424	0.6322
		time x analgesic	F (3, 24) = 0.3155	0.8140

Supplementary Table 8. Statistics for data of figure S4

Figure	Statistic model	Effects	F (DFn, DFd)	p-value
2a	Two Way ANOVA	tissue x analgesic	F (2, 35) = 0.018869	0.9815
		tissue	F (2, 35) = 206.6	<0.0001*
		analgesic	F (1, 38) = 0.3779	0.5427
2b	Unpaired t-Test	t= 0.9080, df =12	2.940, 6, 6	0.3817
	Unpaired t-Test (length)	t=1.216, df=12	1.355, 6, 6	0.2472
	Kolmogorov-Smirnov test (width)			0.5921
2c	Two Way ANOVA	tissue x analgesic	F (2, 32) = 0.1632	0.8509
		tissue	F (2, 32) = 321.4	<0.0001*
		analgesic	F (1, 32) = 2.505	0.1234
2e	Unpaired t-Test	t=1.726, df=11	1.045, 6, 5	0.112
	Unpaired t-Test (length)	t=1.025, df=11	1.547, 5, 6	0.3274
	Kolmogorov-Smirnov test (width)			0.0699
