# Supplementary material for: Improving pain management for murine orthotopic xenograft models of acute lymphoblastic leukemia
Source: Lab Anim (NY). 2025 Oct 8;54(11):313–20. doi: 10.1038/s41684-025-01615-3 (PMC12575361; doi:10.1038/s41684-025-01615-3)
Supplement: Supplementary file 2 — Reporting Summary [file 41684_2025_1615_MOESM2_ESM.pdf]

nature portfolio

Corresponding author(s): Simone Kumstel

Last updated by author(s): Mar 24, 2025

Reporting Summary

Nature Portfolio wishes to improve the reproducibility of the work that we publish. This form provides structure for consistency and transparency in reporting. For further information on Nature Portfolio policies, see our Editorial Policies and the Editorial Policy Checklist.

Statistics

For all statistical analyses, confirm that the following items are present in the figure legend, table legend, main text, or Methods section.

n/a	Confirmed	
☐	☒	The exact sample size (n) for each experimental group/condition, given as a discrete number and unit of measurement
☐	☒	A statement on whether measurements were taken from distinct samples or whether the same sample was measured repeatedly
☐	☒	The statistical test(s) used AND whether they are one- or two-sided<br>*Only common tests should be described solely by name; describe more complex techniques in the Methods section.*
☐	☒	A description of all covariates tested
☐	☒	A description of any assumptions or corrections, such as tests of normality and adjustment for multiple comparisons
☐	☒	A full description of the statistical parameters including central tendency (e.g. means) or other basic estimates (e.g. regression coefficient) AND variation (e.g. standard deviation) or associated estimates of uncertainty (e.g. confidence intervals)
☐	☒	For null hypothesis testing, the test statistic (e.g. F, t, r) with confidence intervals, effect sizes, degrees of freedom and P value noted<br>*Give P values as exact values whenever suitable.*
☒	☐	For Bayesian analysis, information on the choice of priors and Markov chain Monte Carlo settings
☒	☐	For hierarchical and complex designs, identification of the appropriate level for tests and full reporting of outcomes
☐	☒	Estimates of effect sizes (e.g. Cohen's d, Pearson's r), indicating how they were calculated

Our web collection on statistics for biologists contains articles on many of the points above.

Software and code

Policy information about availability of computer code

Data collection	Data for behavioral parameters was collected in microsoft exel, night owl imaging data was collcted via Indigo software (Berthold Technologies, version 1.04)
Data analysis	Data analysis was performed with GraphPaf Prism 8.4.3 (GraphPad Software, San Diego, CA, USA). The RELSA Algorith was calculted with the R-Package (https://talbotsr.com/RELSA/index.html).

For manuscripts utilizing custom algorithms or software that are central to the research but not yet described in published literature, software must be made available to editors and reviewers. We strongly encourage code deposition in a community repository (e.g. GitHub). See the Nature Portfolio guidelines for submitting code & software for further information.

Data

Policy information about availability of data

All manuscripts must include a data availability statement. This statement should provide the following information, where applicable:

- Accession codes, unique identifiers, or web links for publicly available datasets
- A description of any restrictions on data availability
- For clinical datasets or third party data, please ensure that the statement adheres to our policy

The data of the present study are available with the supplementary information of the article. Additional information is available on request from the corresponding author.

Research involving human participants, their data, or biological material

Policy information about studies with human participants or human data. See also policy information about sex, gender (identity/presentation), and sexual orientation and race, ethnicity and racism.

Reporting on sex and gender	no human data in the present study
Reporting on race, ethnicity, or other socially relevant groupings	*Please specify the socially constructed or socially relevant categorization variable(s) used in your manuscript and explain why they were used. Please note that such variables should not be used as proxies for other socially constructed/relevant variables (for example, race or ethnicity should not be used as a proxy for socioeconomic status).*<br>*Provide clear definitions of the relevant terms used, how they were provided (by the participants/respondents, the researchers, or third parties), and the method(s) used to classify people into the different categories (e.g. self-report, census or administrative data, social media data, etc.)*<br>*Please provide details about how you controlled for confounding variables in your analyses.*
Population characteristics	*Describe the covariate-relevant population characteristics of the human research participants (e.g. age, genotypic information, past and current diagnosis and treatment categories). If you filled out the behavioural & social sciences study design questions and have nothing to add here, write "See above."*
Recruitment	*Describe how participants were recruited. Outline any potential self-selection bias or other biases that may be present and how these are likely to impact results.*
Ethics oversight	*Identify the organization(s) that approved the study protocol.*

Note that full information on the approval of the study protocol must also be provided in the manuscript.

Field-specific reporting

Please select the one below that is the best fit for your research. If you are not sure, read the appropriate sections before making your selection.

☒ Life sciences ☐ Behavioural & social sciences ☐ Ecological, evolutionary & environmental sciences

For a reference copy of the document with all sections, see nature.com/documents/nr-reporting-summary-flat.pdf

Life sciences study design

All studies must disclose on these points even when the disclosure is negative.

Sample size	14 mice (7 male /7 female) received respectively the human ALL precursor SEM- cell line or the RS4;11 cell line. In the RS4;11 model, three male and four female mice received metamizole, while four male and three female mice were assigned to the tramaodol treatment group. In the RS4;11 model, three male and four female mice received metamizole, while four male and three female mice were assigned to the tramadol treatment group.
Data exclusions	One male mouse in the SEM cell-induced model did not receive any analgesia since the mouse had to be euthanized immediately due to abnormal respiratory breathing
Replication	The experment was performed first with the RS4; 11 cell line, and afterwards with the SEM cells.
Randomization	The NSG mice were randomized matching the gender of the mice into the different analgesic groups.
Blinding	The researchers were blinded for the analysis of the mouse grimace scale. Blinding of the mice for the analgesic gruops was not possible due to the yellow colour of metamizole.

Reporting for specific materials, systems and methods

We require information from authors about some types of materials, experimental systems and methods used in many studies. Here, indicate whether each material, system or method listed is relevant to your study. If you are not sure if a list item applies to your research, read the appropriate section before selecting a response.

Materials & experimental systems

n/a	Involved in the study	
☒	☐	Antibodies
☐	☒	Eukaryotic cell lines
☒	☐	Palaeontology and archaeology
☐	☒	Animals and other organisms
☒	☐	Clinical data
☒	☐	Dual use research of concern
☒	☐	Plants

Methods

n/a	Involved in the study	
☒	☐	ChIP-seq
☒	☐	Flow cytometry
☒	☐	MRI-based neuroimaging

Eukaryotic cell lines

Policy information about cell lines and Sex and Gender in Research

Cell line source(s)	GFP- and enhanced firefly luciferase (ffLuc)-transduced human precursor ALL cell lines SEM and RS4;11 were provided by Prof. Irmela Jeremias (Helmholtz Center Munich, Germany).
Authentication	Cell lines were regularly checked for authenticity using cell surface marker flow cytometry
Mycoplasma contamination	Cell lines were regularly checked for mycoplasma contamination using the MycoAlert kit (Lonza)
Commonly misidentified lines (See ICLAC register)	Not affected

Animals and other research organisms

Policy information about studies involving animals; ARRIVE guidelines recommended for reporting animal research, and Sex and Gender in Research

Laboratory animals	28 immune-deficient NOD.Cg-Prkdcscid Il2rgtm1Wjl/SzJ mice (NSG) were used in the study 13-20 weeks old.
Wild animals	no wild animals were included in this studie
Reporting on sex	14 mice (7 male /7 female) received respectively the human ALL precursor SEM- cell line or the RS4;11 cell line. In the RS4;11 model, three male and four female mice received metamizole, while four male and three female mice were assigned to the tramaodol treatment group. In the RS4;11 model, three male and four female mice received metamizole, while four male and three female mice were assigned to the tramadol treatment group.
Field-collected samples	The samples were collected in the laboratory only.
Ethics oversight	The local authority Landesamt für Landwirtschaft, Lebensmittelsicherheit und Fischerei Mecklenburg-Vorpommern approved the animal experiments (7221.3-1-63/20-5).

Note that full information on the approval of the study protocol must also be provided in the manuscript.

Plants

Seed stocks	No plants were used
Novel plant genotypes	*Describe the methods by which all novel plant genotypes were produced. This includes those generated by transgenic approaches, gene editing, chemical/radiation-based mutagenesis and hybridization. For transgenic lines, describe the transformation method, the number of independent lines analyzed and the generation upon which experiments were performed. For gene-edited lines, describe the editor used, the endogenous sequence targeted for editing, the targeting guide RNA sequence (if applicable) and how the editor was applied.*
Authentication	*Describe any authentication procedures for each seed stock used or novel genotype generated. Describe any experiments used to assess the effect of a mutation and, where applicable, how potential secondary effects (e.g. second site T-DNA insertions, mosiacism, off-target gene editing) were examined.*
